# Induction of a local muscular dystrophy using electroporation in vivo: an easy tool for screening therapeutics

**DOI:** 10.1038/s41598-020-68135-7

**Published:** 2020-07-09

**Authors:** Aline Derenne, Alexandra Tassin, Thuy Hang Nguyen, Estelle De Roeck, Vincianne Jenart, Eugénie Ansseau, Alexandra Belayew, Frédérique Coppée, Anne-Emilie Declèves, Alexandre Legrand

**Affiliations:** 1 Department of Metabolic and Molecular Biochemistry, Research Institute for Health Sciences and Technology, University of Mons, Mons, Belgium; 2 Department of Respiratory Physiology, Pathophysiology and Rehabilitation, Research Institute for Health Sciences and Technology, University of Mons, Mons, Belgique

**Keywords:** Animal disease models, Musculoskeletal models, Model vertebrates, Biological models, Experimental organisms, Gene delivery, Gene expression analysis, Antisense oligonucleotide therapy, Expression systems, Gene delivery, Nucleic-acid therapeutics, Oligo delivery, Biological techniques, Biotechnology, Molecular biology

## Abstract

Intramuscular injection and electroporation of naked plasmid DNA (IMEP) has emerged as a potential alternative to viral vector injection for transgene expression into skeletal muscles. In this study, IMEP was used to express the *DUX4* gene into mouse *tibialis anterior* muscle. *DUX4* is normally expressed in germ cells and early embryo, and silenced in adult muscle cells where its pathological reactivation leads to Facioscapulohumeral muscular dystrophy. *DUX4* encodes a potent transcription factor causing a large deregulation cascade. Its high toxicity but sporadic expression constitutes major issues for testing emerging therapeutics. The IMEP method appeared as a convenient technique to locally express *DUX4* in mouse muscles. Histological analyses revealed well delineated muscle lesions 1-week after *DUX4* IMEP. We have therefore developed a convenient outcome measure by quantification of the damaged muscle area using color thresholding. This method was used to characterize lesion distribution and to assess plasmid recirculation and dose–response. DUX4 expression and activity were confirmed at the mRNA and protein levels and through a quantification of target gene expression. Finally, this study gives a proof of concept of IMEP model usefulness for the rapid screening of therapeutic strategies, as demonstrated using antisense oligonucleotides against *DUX4* mRNA.

## Introduction

Electroporation (EP), also named electro-transfection, is a non-viral method allowing an enhanced cellular uptake of various type of exogenous molecules such as DNA, RNA, proteins or chemicals. The procedure is based on the application of short electric pulses that transiently permeabilize cell membrane permitting cellular and nuclear entrance of large particles^1–4^. Neumann et al. were the first to report in 1982 that pulsed electric fields could efficiently introduce linear or circular DNA into mouse lyoma cells in culture^5^. The mechanisms by which EP facilitates DNA transport across cell membrane, cytoplasm and nuclear membranes are still debated. Electric pulses induce electrophoretic forces that facilitate migration of charge-carrying molecules, such as naked DNA plasmids (pDNA), maximizing their interaction with cell membranes^6^. Cells exposed to an electrical field present a change in transmembrane potential. At a critical threshold value, a re-orientation of membrane phospholipids occurs, leading to the formation of small hydrophilic openings called electropores. These breakdowns are reversible and allow water, ions and membrane-impermeable molecule flow^2,3,7,8^. By this way, pDNA can enter into cells and its encoded transgene can be expressed. However, recent studies demonstrated the importance of endocytosis pathways (both clathrin- and caveolin-mediated endocytosis) for DNA internalization following EP^3,4,9,10^, notably in mouse muscles^11^. Since its discovery, numerous advances were made in the field and EP was applied with success both in vitro and in vivo in various cell and tissue types. Today, EP has many biomedical applications. Most studies are related to anticancer drug delivery, also called electrochemotherapy, using intratumoral injection coupled with EP to enhance cellular uptake of a therapeutic agent presenting high intrinsic toxicity but low plasma membrane permeability^12–14^. Besides its direct clinical application in the field of cancer, EP is broadly used as a gene delivery tool^15^ that can be applied either for vaccination^16–20^, immunotherapy (especially in cancer applications^21,22^), gene therapy^23–26^, genome editing^27,28^ and generation of induced pluripotent stem cells^29,30^. Number of phase I and II clinical trials are underway or have been completed, demonstrating the safety and efficacy of this procedure^3^. EP has several advantages compared to other tools for gene delivery. Indeed, pDNA are easy to modify and prepare, inexpensive to produce in large scale, and may be administered multiple times without significant inflammation or immune response. pDNA are injected as “naked” molecules meaning that no additional chemicals are associated, limiting risks and undesirable side effects^1,2^. Unlike viral vectors, there is no safety considerations^1,31^. EP procedure is relatively simple and easy to set up and does not need expensive instrumentation. This procedure allows increased levels of gene transfer and expression, nearing those of viral vector, and is applicable from cells transfection to drug and therapeutic gene delivery into living tissues from rodents to humans^2,3^.

Skeletal muscles constitute attractive targets for gene therapy, due to their accessibility and high vascularization. Notably, skeletal muscles were used for systemic delivery of therapeutic protein such as erythropoietin^32^, coagulation factors^33^ or anti-inflammatory cytokines^34^. Muscle EP protocols, first described in^35^, have been optimized over time to improve transfection efficacy and transgene expression level^1,36–40^. In the context of muscle dystrophies, EP methodology was explored as a potential route for treatment. EP was notably reported as efficient to transduce constructs encoding dystrophin in the *mdx* mouse and dog models of Duchenne Muscular Dystrophy (DMD)^41,42^. Contrary to DMD, resulting from the loss of dystrophin, Facioscapulohumeral muscular dystrophy (FSHD)^43^ is a gain of function disease caused by the inappropriate expression in skeletal muscle of *DUX4,* a gene normally only expressed in germline and early embryogenesis^44–55^. The *DUX4* gene encodes a transcription factor that deregulates a large molecular network^53–60^. However, the precise mechanisms by which DUX4 leads to clinical symptoms still must be clarified. Even though various therapeutic strategies are emerging, there is currently no curative treatment for FSHD. Several drug-based therapies aiming either for muscle improvement (anti-inflammatory approach^61^, β2-adrenergic agonists^62–65^, antioxidants^66^) or inhibition of *DUX4* expression such as mitogen-activated protein kinase inhibitors^67^ (Losmapimod, Fulcrum therapeutics, NCT04003974 ) are investigated in clinical trials^68,69^. In parallel, gene therapy has been explored to reduce or avoid DUX4 protein expression and/or activity by controlling *D4Z4* locus methylation^70,71^, or to silence *DUX4* mRNA. Among those, antisense oligonucleotides (AOs) and siRNA targeting the *DUX4* mRNA and preventing its translation have been developed and successfully tested in vitro and in vivo^54,72–75^. Development of in vivo proof-of-concept studies for emerging therapies are now required as a next step towards clinical trials. Several hurdles such as DUX4 toxicity and its stochastic low expression have made the generation of an animal model recapitulating all the pathophysiological aspects of FSHD very challenging. FSHD-like mouse models have now been described, each of them possessing their own advantages and limits^59,76–82^. Some of these mouse models allow inducible conditional DUX4 expression, bypassing DUX4 high toxicity during embryonic development and enabling mice to grow up and develop muscular dystrophy^78–80,83^. These models open new ways to investigate molecular mechanisms leading to FSHD symptoms. However, the use of inducible transgenic models is often time consuming and costly. It is especially an issue in the current context of FSHD where high throughput molecule screenings are required to identify new potential therapeutics. In the present study, we describe a convenient in vivo model of DUX4 local muscle expression using an EP procedure. This model is simple, unexpansive, reproducible and associated with an easy read out that facilitates quantitative analysis. Therefore, this model can be useful at the forefront for high throughput therapeutic screening.

## Results

### Hyaluronidase pre-treatment improves gene expression following naked DNA injection and electroporation in vivo

**Figure 1:**
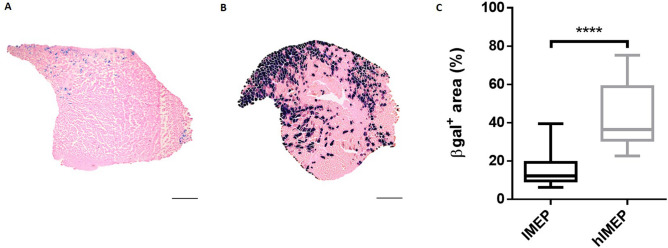
Hyaluronidase pre-treatment improves β-galactosidase expression in mouse *Tibialis Anterior* muscle (TA). (**A**,**B**) Representative sections of TA electroporated (**A**) without hyaluronidase pre-treatment (IMEP) or (**B**) with hyaluronidase pre-treatment 2 h before the electroporation procedure (hIMEP). TA muscles were injected by IMEP or hIMEP with 40 µg of *pCMV-lacZ* reporter plasmid. TA were harvested 1-week post-injection and cryosections stained with X-gal (blue) and counterstained with Eosin (pink). Scale 500 µm. (**C**) Percentage of surface area expressing β-galactosidase (β-gal^+^) quantified by color thresholding using ImageJ. Data are represented as boxplots, ****p < 0.0001 Mann–Whitney Rank Sum Test; n = 4 for each group. The graph was generated using GraphPad Prism 6.01.

Mouse TA muscles were injected with the *pCMV-lacZ* reporter plasmid and then electroporated (IMEP procedure). In order to determine whether muscle pre-treatment with hyaluronidase (which digests hyaluronic acid, a major constituent of the extracellular matrix) could modify gene electroporation efficacy, naked DNA injection was preceded (hIMEP group) or not (IMEP group) by an intramuscular injection (IM) of hyaluronidase. TA muscles were harvested 7 days after injection. The reporter expression level was evaluated by X-gal staining of β-galactosidase activity on cryosections from proximal, medial and distal muscle regions and averaged for each group. As observed in Fig. 1, hyaluronidase pre-treatment significantly improved gene electroporation efficiency as shown by the increased β-galactosidase-positive (β-gal^+^) muscle area (Fig. 1A–C). Quantification of β-gal^+^ muscle surface showed a three-fold increase in mouse TA pre-treated with hyaluronidase, with median value of 36.4% (*p* < 0.001, hIMEP vs IMEP, Fig. 1C). The hIMEP procedure was therefore applied in the next steps of the study.

### TA electroporation with DUX4-expression plasmid induces readily quantifiable muscle lesions

Because of the known DUX4 toxicity in human FSHD muscles, we first checked whether a DUX4 local expression induced by the hIMEP procedure could impact muscle structure in mice. To this aim, the TA muscles were injected and electroporated with *pCIneo-DUX4* expression plasmid using the hIMEP procedure. The empty *pCIneo* plasmid was used as negative control. In both conditions, a simultaneous injection of the *pCMV-lacZ* reporter vector was performed to facilitate the location of muscle areas having incorporated the transgenes. Mice were sacrificed 1 week later and TA muscles were harvested, quickly frozen, cryosectioned and stained with X-gal to detect reporter gene activity, or with Hematoxylin–Eosin–Heidenhain blue (HEB) for histological evidence of muscle damage.

**Figure 2:**
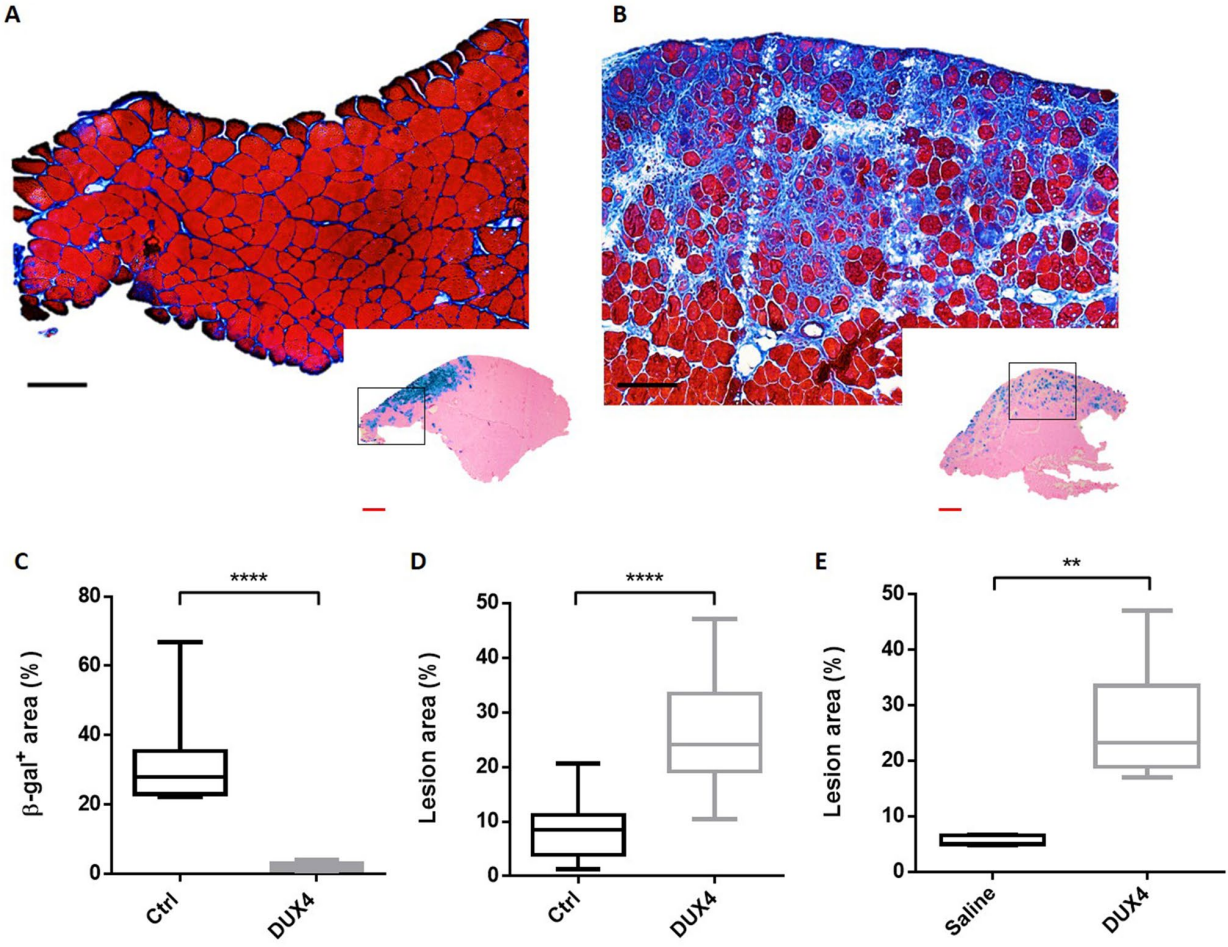
hIMEP of DUX4-expression plasmid induces muscle lesions. (**A**,**B**) Representative sections of TA electroporated with 10 µg of *pCMV-lacZ* and 40 µg of (**A**)* pCIneo* or (**B**) *pCIneo-DUX4* plasmids. TA muscles were harvested 1-week post injection and cryosections from distal, medial and proximal regions stained with X-gal to assess β-gal^+^ areas (small pictures). Adjacent sections were stained with HEB coloration for muscle damages evaluation (large pictures). Scales 50 µm (red) and 100 µm (black). (**C**,**D**) Percentage of surface area (**C**) expressing β-galactosidase (β-gal^+^) and (**D**) damaged in mouse TA 1-week post hIMEP using 10 µg of *pCMV-lacZ* concomitant with 40 µg of *pCIneo* (Ctrl) or *pCIneo-DUX4* (DUX4) plasmids. β-gal^+^ and lesion area percentage were evaluated on total section stained with X-gal or HEB respectively, and quantified by color thresholding using ImageJ. (**E**) Recirculation test. Both TA from same mouse were electroporated, one using 40 µg of *pCIneo-DUX4*, the other saline solution. One week after hIMEP, lesion area was evaluated on HEB stained TA cryosections by color thresholding using ImageJ. All results are presented as boxplots, **p < 0.01 and ****p < 0.0001 Mann–Whitney Rank Sum Test; n = 5 for each group except in (E) n = 2. Graphs were generated using GraphPad Prism 6.01.

One-week post-injection, TA muscles electroporated with the control plasmid presented a normal histological structure with peripheral nuclei in geometric fibres surrounded by a thin layer of endomysial extracellular matrix, as shown by HEB staining (Fig. 2A). A restricted area exhibiting some smaller fibres, central nuclei and a slight focal inflammatory infiltrate around the site of injection (β-gal^+^ area) was sometimes observed (data not shown). X-gal staining in control TA confirms electroporation efficiency as shown by the presence of grouped β-gal^+^ fibres (Fig. 2A, Box). In contrast, TA muscles electroporated with the DUX4-expression plasmid showed a large damaged region containing atrophic fibres with high size variability, some regenerating fibres characterized by central nuclei, a marked inflammatory infiltrate and an accumulation of conjunctive tissue (fibrosis) (Fig. 2B; SI Fig. S1 online). Interestingly, X-gal staining in adjacent sections showed a lower number of β-gal^+^ myofibres with a different spatial distribution as compared to TA muscles injected with the control plasmid (Fig. 2B, Box). Indeed, a median value of 2.1% of the total muscle surface was positive for β-gal in the DUX4 expression group against 27.97% in the control group (*p* < 0.001, Fig. 2C). As expected, lesion area quantification on HEB-colored total cryosections indicated that the percentage of damaged surface area was significantly higher in TA muscles injected with the DUX4-expression plasmid as compared to the control group (*p* < 0.001) with a median value of 25.1 and 8.5%, respectively (Fig. 2D).

We also investigated the plasmid recirculation potential following hIMEP. Indeed, we were wondering whether the recirculation of the DUX4 expression plasmid would be sufficient to cause transfection of the contralateral TA resulting in quantifiable lesions. To this aim, both TA muscles from the same mouse were electroporated, one using *pCIneo-DUX4*, the other one using saline solution. One week after hIMEP, quantification of the damaged area on HEB-stained cryosections showed no detectable lesion in the saline-injected TA, in contrast to the contralateral TA injected with the DUX4-expression plasmid which exhibited a significant lesion area (Fig. 2E). This suggested that no plasmid recirculation occurred, allowing an independent treatment of both TA from the same animal.

### Dose–response analysis

**Figure 3:**
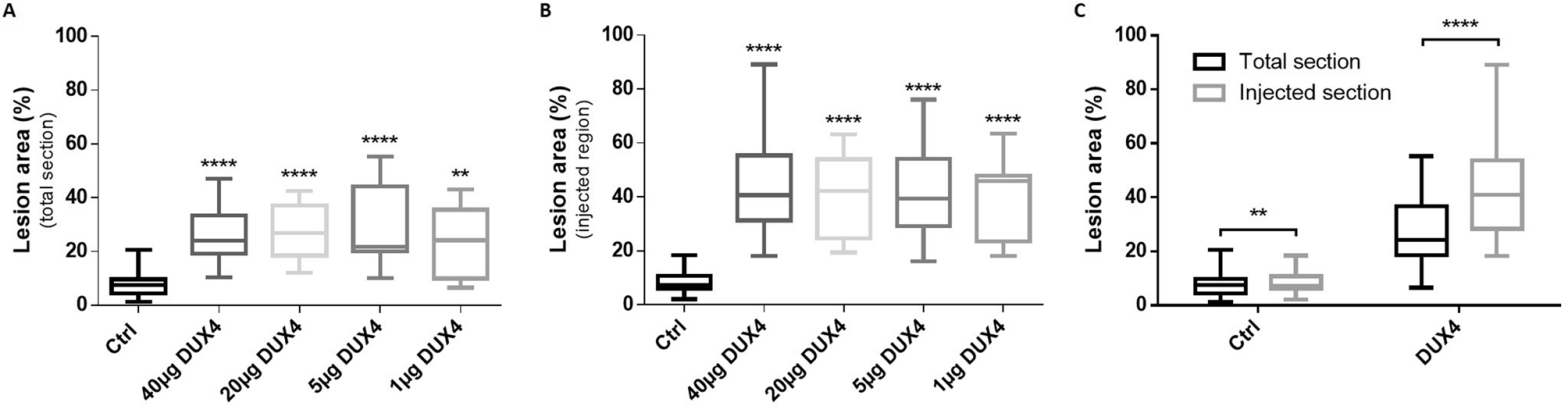
Dose–response of muscle lesion area in mouse TA 1-week post hIMEP procedure. (**A**,**B**) Lesion area percentage was evaluated (**A**) on total cryosection or (**B**) in the injected area (defined by β-gal^+^ region) from distal, medial and proximal part of TA electroporated with different doses of control or DUX4-expression plasmid and 10 µg of *pCMV-lacZ*. Sections were stained with HEB and lesions were quantified by color thresholding using ImageJ. Control groups (Ctrl) include 1-, 20-, 40-µg *pCIneo*-injected groups as there was no statistical difference among them (one-way ANOVA followed by Dunn’s post hoc test, p = 0.959 and 0.153 for total and injected region quantifications respectively, NS). (**C**) Comparison of injured area percentages quantified on total section (black) or in injected area (defined by the β-gal^+^ region) (grey). DUX4 groups include 1-, 5-, 20-, 40-µg *pCIneo-DUX4*-injected groups as there was no statistical difference among them (one-way ANOVA followed by Dunn’s post hoc test, p = 0.894 and 0.957 for total and injected regions respectively, NS). All results are presented as boxplots, ****p < 0.0001, **p < 0.01, (vs ctrl (**A**,**B**) or as indicated (**C**)). (**A**,**B**) One-way ANOVA on the ranks followed by Dunn’s post hoc test; n = 3, except 5 µg DUX4 (n = 4), 40 µg DUX4 (n = 5) and Ctrl (n = 11). (**C**) Wilcoxon signed rank test; n = 11 (ctrl) or 15 (DUX4). Graphs were generated using GraphPad Prism 6.01.

A dose–response analysis was performed by electroporating mouse TA muscles with increasing doses of the DUX4-expression plasmid or control plasmid, concomitantly with the *pCMV-lacZ* reporter vector. The quantification of muscle damages (HEB staining) was (1) reported either to total muscle section or (2) to the injected area, corresponding to the β-gal^+^ region. Regardless of the quantification method, no statistical difference was observed following the injection of different doses of the control plasmid (1–20–40 µg *pCIneo*; one-way ANOVA on the ranks followed by Dunn’s post hoc test, *p* = 0.959 and 0.153 in total and injected area, respectively, data not shown). The data from these three control groups were therefore pooled in a single control group (Fig. 3). Regarding the muscle damage quantification reported to total section (Fig. 3A), a significant increase of damaged surface between the control group and all DUX4 groups was demonstrated (*p* < 0.01). Similar results were obtained when the injected area was the only area considered for the quantification (Fig. 3B). However, there was no significant difference in the percentage of damaged area obtained after injection of increasing doses of *pCIneo-DUX4* using either quantification system (*p* = 0.894 and 0.957, reported to total section or to injected area, respectively). Data from the four DUX4 expression groups have thus been grouped (in Fig. 3C) to compare results obtained from both quantification methods. As expected, the percentage of damaged muscle surface area was higher when quantified within the injected region, as compared to the percentage calculated on total muscle section. This difference is highly statistically relevant in the DUX4 group (*p* < 0.0001) and significant in the control groups (*p* = 0.01) where the altered muscle area was limited to 7.4% of the total muscle section and to 7.3% when calculated in the injected region (Fig. 3C).

### Distribution of the lesions through *Tibialis Anterior* muscles

**Figure 4:**
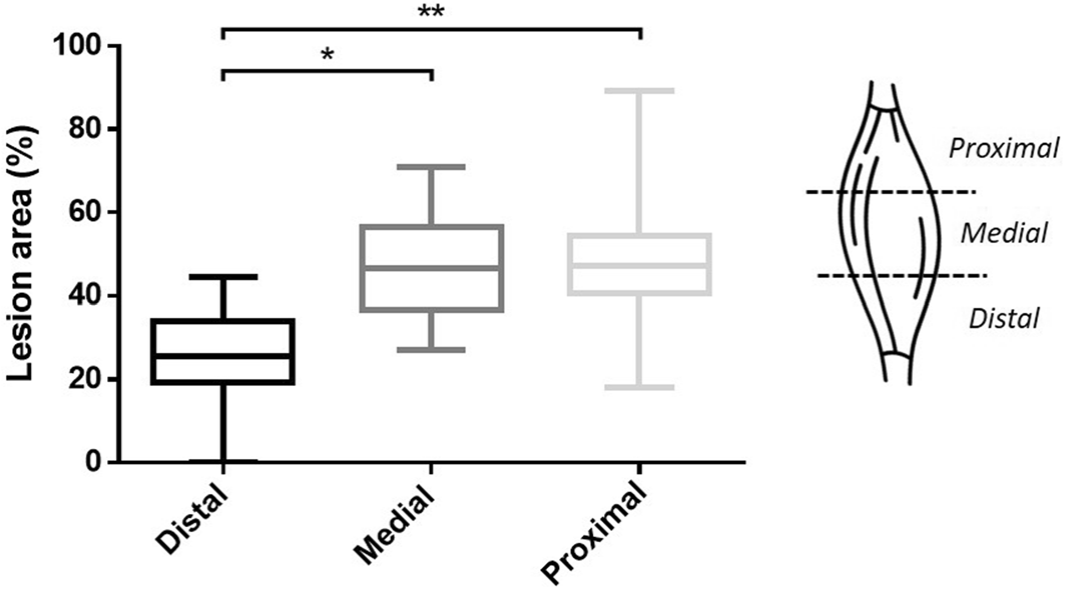
Lesion distribution through TA regions. Mouse TAs were electroporated with different doses of *pCIneo-DUX4* (1, 5,10, 20 and 40 µg) and 10 µg of *pCMV-lacZ*. Muscle damaged area 1 week following TA hIMEP was evaluated in the injected area (β-gal^+^) of HEB stained cryosections from distal, medial and proximal regions of TA (color thresholding, ImageJ). Since no statistical difference was observed between tested doses considering each region individually, data were respectively pooled to form single proximal, medial and distal groups (data not shown, One-way Anova, p = 0.871, 0.821 and 0.395 in proximal, medial and distal region respectively). All data are presented as boxplots, *p < 0.05 and **p < 0.01. Friedman Repeated Measures Analysis of Variance on Ranks; n = 15 for each group. Graphs were generated using GraphPad Prism 6.01.

In order to investigate DUX4 expression distribution through TA following hIMEP with *pCIneo-DUX4*, muscle damages were quantified in 3 different TA regions (proximal, medial and distal region). Since no statistically significant difference was observed between tested doses considering each region individually (One way Anova, *p* = 0.871, 0.821 and 0.395 in the proximal, medial and distal region, respectively, data not shown), data corresponding to the 1-, 5-, 20-, 40 µg DUX4 plasmid groups were respectively pooled for each muscle region. As illustrated in Fig. 4, at 7 days post-injection, the percentage of damaged muscle area in the proximal and medial part of the TA were not statistically different, with respective medians of 47.11% and 46.69% (*p: NS*, Friedman Repeated Measures Analysis of Variance on Ranks). Regarding the distal muscle part, the percentage of lesion was significantly lower than in the other muscle regions (*p* < 0.05) with a median of 25.6% (Fig. 4). Considering these results, the subsequent analyses were based on the proximal and medial TA parts to minimize variability.

### Time-course analysis

**Figure 5:**
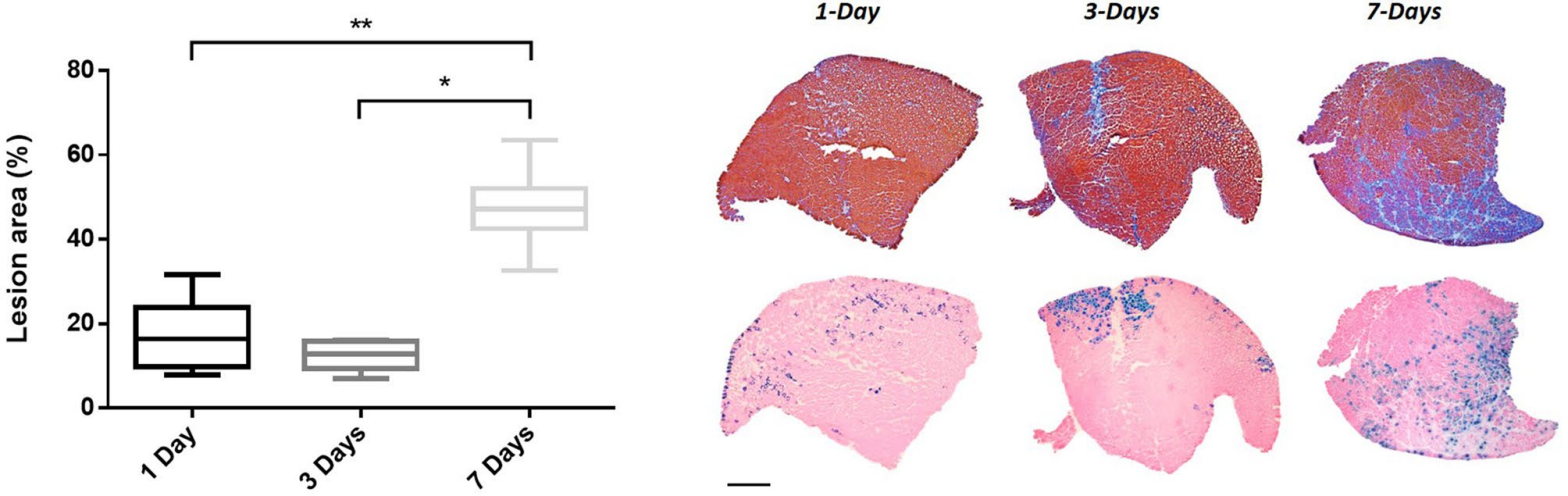
Time-course analysis. TA muscles were electroporated with 1 µg of *pCIneo-DUX4* and 10 µg of *pCMV-LacZ*. Lesion area was evaluated 1-, 3- or 7-days after hIMEP in the injected area (β-gal^+^) on HEB stained cryosections of medial and proximal TA regions (color thresholding, ImageJ). Results are presented as boxplots, *p < 0.05 and **p < 0.01. One-way ANOVA on the ranks followed by Dunn’s post hoc test; n = 3 for each group. Scale 1 mm. The graph was generated using GraphPad Prism 6.01.

The development of the TA lesions was evaluated 1-, 3- and 7-days after the hIMEP procedure by using 1 µg of the DUX4-expression plasmid and a concomitant injection of *pCMV-lacZ* reporter. Muscle damages were then evaluated in TA proximal and medial parts by focusing on the injected area (β-gal^+^ region). At 1- and 3-days after injection, a slight thickening of the extracellular matrix and a straight mark with some mononuclear cells and regenerating fibres (probably corresponding to the injection site) were observed. This led to a global ‘lesion’ percentage defined by HEB staining of 16.3% and 12.9% at 1- and 3-days post-injection, respectively. At 7 days after electroporation, extensive injuries, a marked inflammatory infiltrate and an extracellular matrix expansion were detected. At this time point, damaged surface area represented 47.2% of the injected area (Fig. 5, median value, *p* < 0.05 7-days vs 1- and 3-days).

### Expression of DUX4 and its target genes in hIMEP mice

**Figure 6:**
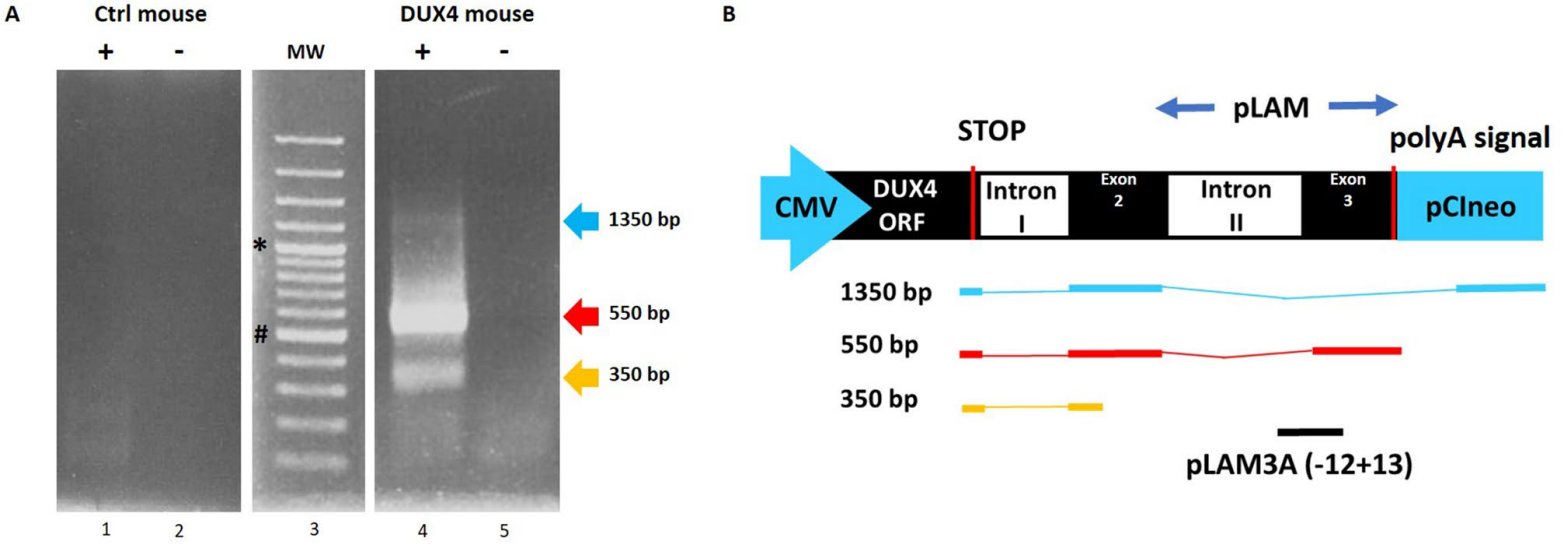
Confirmation of *DUX4* mRNA expression by 3′RACE. (**A**) Nested PCR was used to amplify the 3′ end of *DUX4 *transcripts from total RNA extracted from TA muscles electroporated with 1 µg of control (left panel) or DUX4-expression pDNA (right panel). Representative cropped gels. Lanes 1 and 2 come from one gel; lanes 3, 4 and 5 come from a second gel ran in parallel. Corresponding full-length gels are available in supplementary information (SI Fig. S2 online). 3′RACE were performed 1-, 3- and 7-days post hIMEP. Wells (**−**) show negative controls without retro-transcription. Several fragments were detected (~ 1,350 bp, ~ 550 bp and ~ 350 bp; colored arrows) in each time point (not shown), cloned, sequenced and analyzed to confirm *DUX4* specificity. (**B**) Schematic representation (not to scale) of 3′RACE products analysis. In silico alignment confirmed conservation of vPMO [pLAM3A (− 12 + 13)] target sequence (black line).

**Figure 7:**
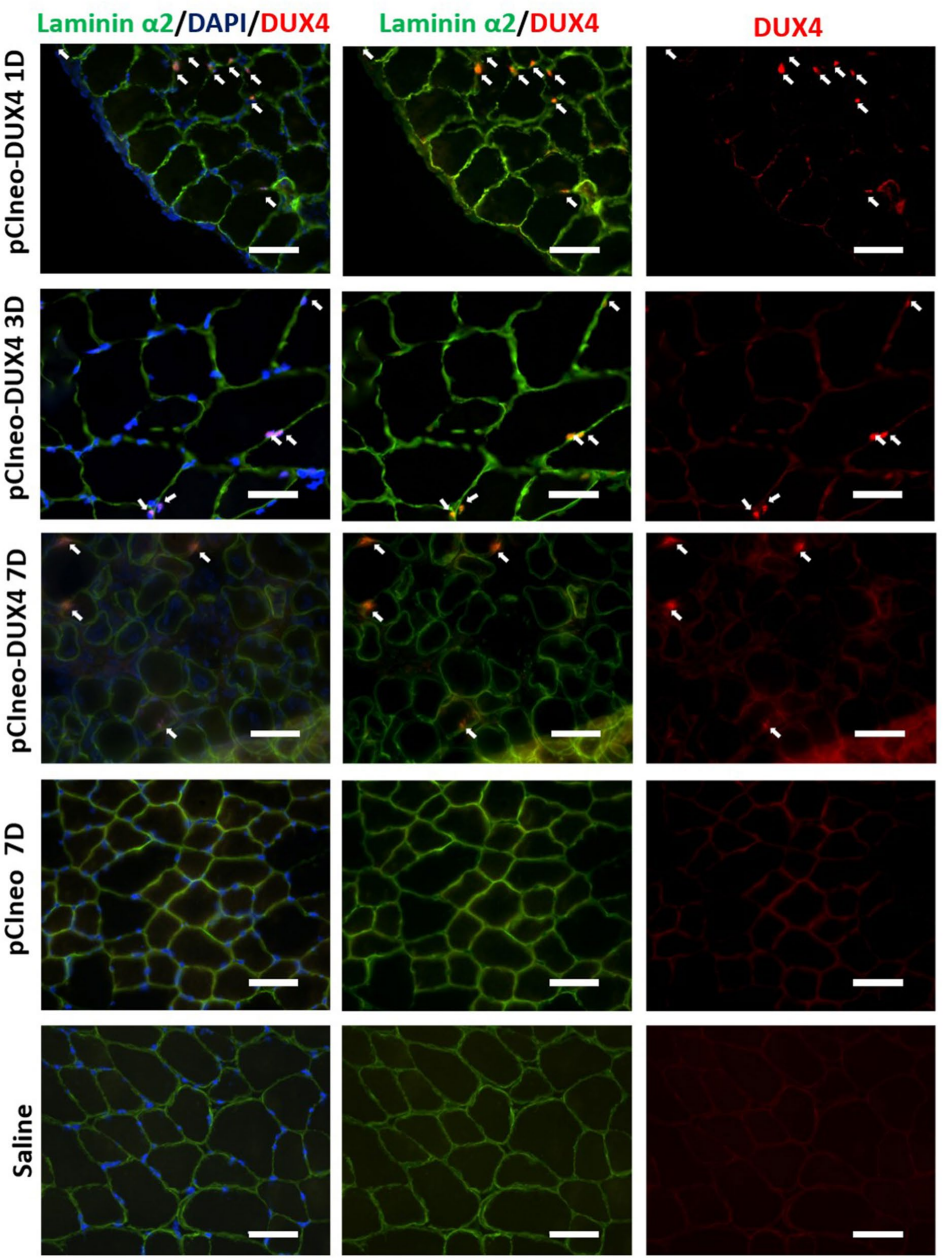
Confirmation of DUX4 protein expression by Immunofluorescence. Mouse TAs were injected by hIMEP with 10 µg *pCMV-lacZ* plasmid concomitantly with 40 µg either *pCIneo-DUX4* or* pCIneo*, or with a saline solution (negative control), as indicated. Cryosections were analyzed 1, 3 and 7 days post-injection by immunofluorescence with antibodies directed against either DUX4 (E5-5) (red) or laminin α2 (green) to stain the myofibre basal lamina (for details, see “Methods”). DAPI was used to visualize nuclei (blue). Pictures were taken with a Nikon Eclipse 80i microscope and merged using NIS-Elements software. Scale bar 50 µm.

**Figure 8:**
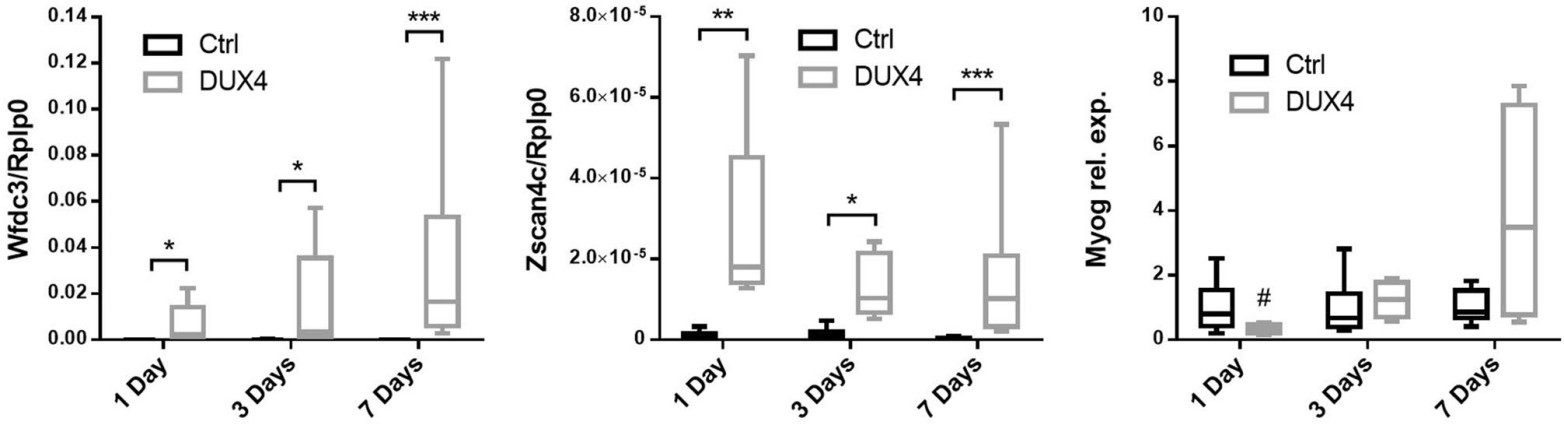
RTqPCR analyses of DUX4 target genes (*Wfdc3, Zscan4c*) and *Myog* expression level in TA muscle. TAs were injected by hIMEP with either 1 µg of control *pCIneo* DNA, 1 µg of *pCIneo-DUX4*- or with 50 µl of saline solution and harvested 1-, 3- or 7-days post injection. Total RNA was extracted with Trizol and qPCRs were performed in duplicates using SYBR Green FastStart Essential DNA Green Master. Analyzes were performed with LightCycler 96 software. At each time, results obtained from control plasmid and saline solution injected groups were pooled to form a single control group, as no statistical difference could be highlighted between them for all tested genes (one-way ANOVA on the ranks followed by Dunn’s post hoc test). Results are presented as fold difference to *Rplp0* for *Wfdc3* and *Zscan4c* and as relative to control for *Myog*. All data are presented as boxplots, *p < 0.05, **p < 0.01, ***p < 0.001 and ^#^p < 0.05 vs DUX4 7 days, One-way ANOVA on the ranks followed by Dunn’s post hoc test n = 14 (Ctrl) or 7 (DUX4) (*Wfdc3* and *Zscan4c*) and n = 8 (ctrl) or 4 (DUX4) (*Myog*). Graphs were generated using GraphPad Prism 6.01.

*DUX4* expression at the mRNA level was investigated by 3′RACE in hIMEP treated mice. In addition, mRNA levels of two DUX4 target genes (*Wfdc3, Zscan4c*)*,* commonly used as biomarkers of DUX4 transcriptional activity^78–80^, were quantified along with the mRNA level of *Myog* a myogenic marker known to be downregulated in FSHD, contrary to *Wfdc3* and *Zscan4c*^84–86^. To this aim, total mRNAs were extracted from mouse TAs treated by hIMEP (1-, 3- and 7-days) using 1 µg of *pCIneo-DUX4* or control plasmid *pCIneo* concomitant with reporter vector *pCMV-lacZ*. The 3′RACE products were separated by electrophoresis on agarose gel and 3 distinct product lengths (~ 1,350 bp, ~ 550 bp and ~ 350 bp) were observed. These fragments were only detected in the DUX4 expression group and at all investigated time points. Interestingly, the 550-bp fragment presented with the highest intensity and frequency (8/12 tested samples), suggesting a higher expression level of the corresponding mRNA as compared to the others. No specific fragment was detected in control mice (Fig. 6; SI Fig. S2 online). All PCR products were cloned, sequenced and analyzed to ensure *DUX4* specificity. We also confirmed, by in silico analysis, presence of the target sequence for antisense oligonucleotide pLAM3A (− 12 + 13) (described in^54,72^) in the 550-bp sequences. DUX4 expression was also evaluated at the protein level by immunofluorescence (Fig. 7) confirming the presence of DUX4-positive nuclei, only in TA muscles injected with *pCIneo-DUX4* at all investigated time-points. RT-qPCR analysis of DUX4 target gene mRNAs highlighted a statistical increase of *Wfdc3* and *Zscan4c* expression at each time point in the DUX4 group compared to the control group (*p* < 0.001). However, there was no statistical difference between time points for these genes. *Myog* mRNA levels were stable in control groups regardless of the time point but significantly decreased at day 1 post hIMEP in the DUX4 expression group. *Myog* expression then increased over time. However, we could not detect any significant difference between DUX4 and control groups at 3- and 7-days post hIMEP (Fig. 8).

### Preliminary evaluation of antisense oligonucleotide injected into DUX4 hIMEP mouse

**Figure 9:**
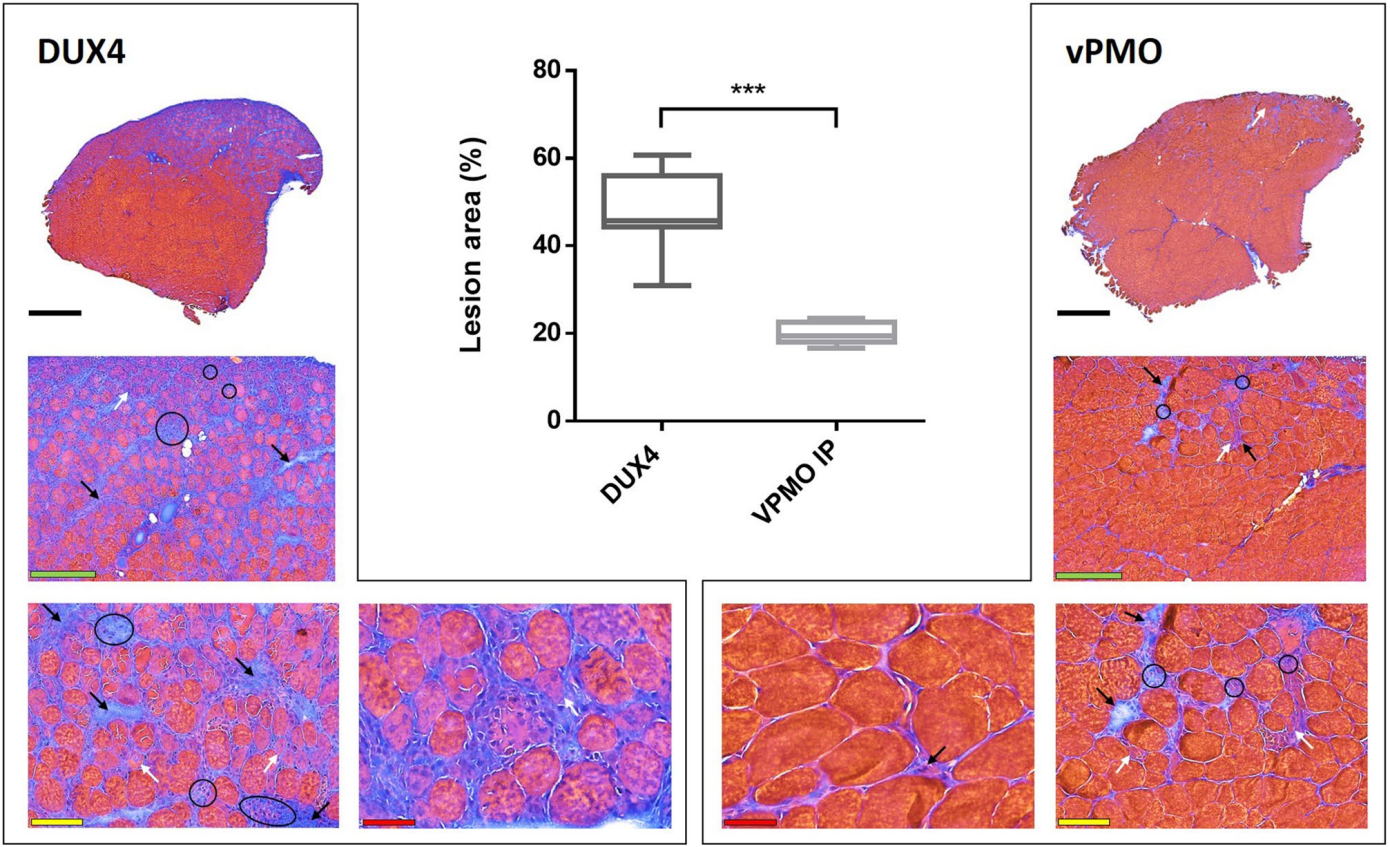
vPMO treatment of mice electroporated with DUX4-expression plasmid decreases TA muscle lesions. TA muscles were injected by hIMEP with 20 µg of *pCineo-DUX4* plasmid. Six hours later, mice received either an intraperitoneal injection of 250 µg of vPMO pLAM3A (− 12 + 13) targeting *DUX4 *mRNA (vPMO group) or no supplemental treatment (DUX4 group). Percentage of lesion area was evaluated 7 days post IMEP by color thresholding using ImageJ on HEB colored cryosections of TA proximal and medial parts. Results are presented as Box plots, ***p < 0,001 by Mann–Whitney Rank Sum Test; n = 4 for each group. The graph was generated using GraphPad Prism 6.01. Black arrows show fibrosis, white arrows atrophic fibres, black circles inflammatory infiltrate. Scales 1 mm (black) 250 µm (green), 100 µm (yellow) and 50 µm (red).

Given that the DUX4 hIMEP mouse model was developed in order to easily test the efficiency of therapeutics in vivo, we investigated antisense oligonucleotide pLAM3A (− 12 + 13) (described in^54,72^) with the vPMO (octa-guanidine conjugated Phosphorodiamidate Morpholino Oligomer) chemistry as a proof-of-concept study. This vPMO silences *DUX4* mRNA translation by interfering with intron II splicing. Its efficiency was previously demonstrated in vitro^54^ and in vivo^72^ in another mouse model^87^. To this aim, mouse TA muscles were electroporated using 20 µg of *pCIneo-DUX4* plasmid by hIMEP and treated 6 h later with a single intraperitoneal injection of 250 µg of vPMO pLAM3A − 12 + 13. Seven days after injection, a highly significant 2.5-fold decrease of histological lesion area was observed in TA muscles of mice treated with the vPMO, as compared to untreated hIMEP DUX4 mice (*p* < 0.001) (Fig. 9).

## Discussion

Non-viral methods for transgene delivery have received increased attentions thanks to their relative safety, simplicity and cost-effectiveness compared to the administration of viral vectors which were found associated with cytotoxicity, inflammation and immune response^1,2^. Initially, the main drawback of naked pDNA gene transfer was its low transfection efficiency limiting its use in fundamental research or therapeutic applications. Indeed, as there are no specific DNA transporters at the surface of mammalian cells, naked plasmids can only be internalized by non-specific endocytosis. As this is a slow process, most of the injected DNA is retained into extracellular space and degraded by endogenous DNAses^88^. Nevertheless, electroporation (EP) was shown to improve transgene uptake and expression both in vitro and in vivo^2,3,7^. Previous studies have demonstrated the importance of various factors influencing efficacy and safety of pDNA EP in several tissues, *inter alia*, skeletal muscles. These factors include electrical parameters (intensity, duration, pulse number and frequency), electrode type and geometry, plasmid features including its length and GC content and finally, additional elements such as the host age or tissue permeability (e.g. normal vs DMD muscle^41^). In the present study, we applied optimized EP parameters according to Mir et al.^89^ who have demonstrated the influence of voltage-to-distance (between both electrodes) ratio, pulse length, number and frequency for muscle EP in vivo. Their observations suggested that 8 pulses of 200 V/cm, 20 ms and 2 Hz was an optimal combination for high transgene expression in mouse skeletal muscle. Those parameters have been largely and successfully used in other studies^1,3,38,40,41,90,91^. Different types of electrode can be used, such as plate electrodes, penetrating needle electrodes or multi-electrode arrays^2,3,38^. Here, we chose to use plate electrodes which have the advantage to apply a large and uniform electrical field^92,93^, to be less invasive and less damaging than the needle one^38,40^. Furthermore, the efficiency of gene transfer essentially depends on the effective DNA electrophoresis and distribution in the area where the electrical field is applied. However, the extracellular matrix (ECM) surrounding the myofibre surface limits a homogenous distribution of pDNA and thereby reduces transfection efficiency. Different enzymes modifying connective tissue permeability such as hyaluronidase or collagenase were tested to improve the distribution of naked pDNA^33,39,40,94–96^. Hyaluronidase is a mucolytic enzyme that depolymerizes *N*-acetyl-hexosamine glycosidic bonds in hyaluronic acid, interfering with its barrier function in the ECM^96^. Intact hyaluronic acid binds water molecules and forms a gelatin-like matrix that provides a structural support for the surrounding tissue. Hyaluronic acid fragments resulting from hyaluronidase digestion are still able to bind water molecules but fail to form a functional viscous gel. Consequently, the density of glycosaminoglycans is reduced, allowing a better DNA electrophoresis, improving its contact with the plasma membrane, thus increasing the transfection rate^95,97^. Consistent with previous studies, we observed an increased reporter gene (*pCMV-lacZ*) expression level when hyaluronidase was injected 2 h before the IMEP procedure. Histological analysis did not reveal any apparent muscle alterations linked to hyaluronidase pre-treatment, consistent with other observations^36,39,94–96^. Moreover, hyaluronidase is currently used in clinical applications^96^ supporting that it is not by itself harmful for tissue.

In this study, the EP procedure was used in order to develop a simple mouse model of DUX4 local expression in skeletal muscle, with the end purpose of rapid therapeutic testing for FSHD. The development of a mouse model recapitulating the full FSHD phenotype and molecular signature is very challenging due to the need for DUX4 precise expression timeframe in the early embryo^98,99^. Attempts at the generation of transgenic mouse models revealed that a tight regulation of DUX4 expression was essential to obtain viable mice able to reach adulthood and to develop a dystrophic phenotype^76,77^. The most recently developed murine models allow a controlled DUX4 expression: two of these are the iDUX4 mouse [2.7]^77^ and its improved version iDUX4pA^78^, both doxycycline-regulated. In addition the FLExDUX4^79,83^ and the TIC-DUX4^80^ mice allow conditional expression of human *DUX4-fl* following Cre recombination. When crossed with the appropriate cre-driver line (tamoxifen-inducible cre line, cre expressed from a muscle-specific promoter), those models allow the appearance of a progressive myopathy, useful for investigations on FSHD pathophysiological mechanisms. However, the use of inducible transgenic models is often time consuming and costly. Therefore, cheap convenient in vivo models, with a local well delineated muscle lesion, although not representing the whole disease complexity, may constitute excellent tools in the frame of initial high-throughput therapeutic screening. Moreover, the IMEP model is customizable. For instance, a construct containing a weaker promoter^100^ could be used in order to mimic a more “physiological” DUX4 expression level. This type of model would be useful to better understand FSHD pathophysiological mechanisms. In addition, the contribution of modifier genes could be similarly investigated. In the present study, we have demonstrated that a single injection of *pCIneo-DUX4* plasmid into mouse TA by hIMEP leads to the development of muscle damage after 7 days. These damages are directly linked to DUX4 expression, as evidenced by the absence of comparable lesions in muscles electroporated in the same conditions with control pDNA. Lesions in muscles injected with DUX4-expression plasmid are characterized by the presence of degenerating fibres with a heterogeneity in size, including numerous small fibres. Large mononuclear cell infiltration, extracellular matrix thickening, fibrosis and central nucleation are also observed as described in other DUX4 studies^59,78–80^. Conversely, control TAs display a normal histology without dystrophic pattern but some of them showed a thin and straight lesion characterized by fibres with central nuclei, sign of regeneration events most probably due to the needle injury caused by the intra-muscular injection. Since our results suggest an absence of significant systemic dissemination of pDNA following hIMEP, both hindlimbs could be used as independent systems, fitting therefore with the animal ethics principle of reduction. The local character of this myopathy model also presents two other advantages. First, only the injected muscle is impaired, so mice do not show any phenotype^59,78–80^. Second, the localized lesion observed 7 days after administration of DUX4 pDNA by hIMEP facilitates therapeutic evaluation. To this aim, we developed an easy and reproducible quantification method to evaluate the percentage of damaged muscle surface area. To ensure the distinction between healthy and injured area, we improved the basic hematoxylin–eosin histological coloration by adding a step of Heidenhain’s blue counterstaining that results in a high blue-pink contrast, easily distinguishable by automatic color thresholding. As expected, the percentage of damaged surface area is significantly higher in the injected area (β-gal^+^) than in the total TA section in DUX4 pDNA-injected mice. Interestingly, in the DUX4 group, we noticed a decrease of the β-gal^+^ surface area and a different spatial distribution of β-gal^+^ fibres which appear more scattered, as compared to the control group. This could be explained by the DUX4 toxicity. Indeed, since both plasmids are co-injected, it is reasonable to hypothesize that most fibres expressing the LacZ plasmid also express DUX4. Due to its high toxicity, the DUX4 protein likely causes fibre degeneration, sarcolemmal damages and a consecutive loss of β-gal signal.

We were not able to highlight a reduction of muscle damage by decreasing DUX4 plasmid doses. If the absence of dose–response is surprising, different hypotheses may be suggested. First, those results are in agreement with the high myotoxic potential of DUX4, even when initially expressed in a limited number of myonuclei, as described previously either in patients^52,55,101,102^ or in FSHD animal models^78–80^. As described by Tassin et al.^55^ and confirmed by Ferreboeuf et al.^102^, the model of “DUX4 nuclear spreading” and the subsequent amplification of the DUX4-induced cascade could partly explain this phenomenon. Second, direct DUX4-induced deregulation pathways are most probably followed by feedback loop processes generating a self-sustaining system involving e.g. oxidative stress and/or inflammation^66,103^. By this way, structural muscle damages observed in our study certainly result from pathophysiological processes that have become independent from the initial DUX4 boost.

Regarding spatial distribution of lesions within the TA muscle, we did not find any significant difference in lesion extent between medial and proximal sections, but a lower percentage of damaged surface area was observed in the distal TA part. This result was unexpected given that the injected transgene could in theory diffuse along the entire myofibres. Moreover, as DUX4 is a transcription factor and harbors nuclear localization signals^104^, it can be transported into several neighboring nuclei in a myotube, as described in previous studies^55,102^. Parameters such as electrode shape and positioning may be involved in inter-regional variation of hIMEP efficiency. Since TA is not cylinder- but cone-shaped, the distances between each muscle region and the electrodes are not identical along the whole muscle. As the distal TA part is located close to the tendon, the contact with electrode and the resulting electrical field was probably lower in this region, despite the use of a conductive gel. Nevertheless, pDNA may theoretically diffuse through the entire myofibre. However, since pDNA is administrated through a single injection, a dilution phenomenon could explain a lower expression in the regions remote from the injection site^55^. In addition, a limited diffusion of nuclear proteins in muscle fibres has already been mentioned^105,106^. The same observations were reported in porcine muscle following EP: in that study transgene expression was limited to the muscle area delineated by the electrical field but no mechanistic hypothesis was proposed^107^. The DUX4 hIMEP procedure is therefore associated to the rapid development of localized muscle lesions. Although this pattern allows to provide an easy read-out for therapeutic screening, the study of pathophysiological mechanisms underlying consequences of DUX4 muscle expression requires a more scattered distribution pattern of the transgene, closer to the one observed in FSHD muscles. This pattern may be obtained by using another route of delivery of naked pDNA. Indeed, the hydrodynamic injection of pDNA via the saphenous vein, first described by Hagstrom et al*.*^108,109^ allowed a more diffuse expression of the transgene in a panel of hindlimb muscles.

Regarding time-course investigations, no damages were observed 1 and 3 days post-hIMEP with DUX4 expression plasmid, while *DUX4* mRNA and protein were already detected. Seven days after the hIMEP procedure, a severe muscle lesion had developed. Although the presence of early damages between the 3rd and the 7th day post-injection cannot be excluded, the period of 7 days seems appropriate to obtain quantifiable and well-delineated muscle lesions consecutive to DUX4 expression. It seems also reasonable to hypothesize that a delay is necessary for the establishment of pathophysiological processes which will ultimately overcome skeletal muscle compensatory mechanisms. Since the expression of DUX4 target genes (“DUX4 footprint genes”) is a good marker of DUX4 protein expression and transcriptional activity^78–80^, DUX4 target gene signature was investigated at the mRNA level to confirm the production of a functional DUX4 protein. As expected, at all-time points, *Wfdc3* and *Zscan4c* expressions were induced in DUX4 pDNA-injected TAs as compared to controls. *Myog*, a marker of muscle regeneration, presented a decreased expression 1-day post-injection. This is similar to results published by other groups showing that *Myog* expression was decreased by DUX4 expression^84–86^. The progressive increase over time is consistent with histological regeneration features (central nuclei) observed 7-days after IMEP. DUX4 protein level was also investigated and as expected given our expression data on *DUX4* mRNA and its target genes, DUX4 protein could be detected at all-time points.

Finally, given the relevance of our mouse model and quantification system in the framework of antisense therapy, a preclinical proof-of-concept study was performed with an antisense oligonucleotide (AO) directed against the *DUX4* mRNA^54,74^. To this aim, we used *pLAM3A *(*− 12* + *13*) AO targeting a splice site in the* DUX4* mRNA 3′UTR region. This AO was previously synthesized with a *2′OMe* chemistry (phosphorothioate backbone) and successfully tested in vitro either on cells overexpressing DUX4 or on FSHD primary myotubes^54^. The same AO synthetized as a *vPMO* (octa-guanidine dendrimer-conjugated vivo morpholino) was used in a preliminary study in vivo showing its ability to decrease *DUX4* mRNA expressed from an AAV vector injected in mouse TA^74^*.* In that study, the vPMO efficiency was monitored through *DUX4* mRNA detection and semi-quantification using 3′RACE. In the present study, we first confirmed that the target sequence of this AO was conserved in the *DUX4* mRNA detected in mouse TA electroporated with the DUX4-expression plasmid. We then tested this vPMO on the DUX4 hIMEP model and demonstrated it could prevent the development of DUX4-induced histological muscle damages.

In conclusion, we have developed a rapid and easy-to-use mouse model of DUX4 local over-expression. Even if the mouse in which TAs are injected with *pCIneo-DUX4* by hIMEP does not recapitulate all the complexity of FSHD pathophysiology, this model has an added value in the forefront of pre-clinical evaluations, particularly in a context in which high throughput therapeutic screening is still necessary. This model could also be applied for the injection of other expression vectors to model various gain-of-function muscular diseases in order to test potential therapeutics.

## Methods

### Ethics statement

All animal experiments met the Belgian national standard requirements regarding animal care and were conducted in accordance with the Ethics and Welfare Committee of the University of Mons. Protocols were approved by the Ethics and Welfare Committee of the University of Mons (reference number LE016/03).

### Animals

Female C57BL/6 mice, aged between 8 and 12 weeks, were purchase from Charles River laboratories (France). Mice were housed in a conventional animal colony and maintained at 35–40% relative humidity with a constant room temperature (21 °C) and natural day/night light cycle (12–12 h). Food and water were provided ad libitum and animals were subjected to an adaptation period of 7 days before starting experiments.

### Plasmids

The commercial reporter plasmid *pCMV-lacZ* encoding the *Escherichia coli* β-galactosidase protein was used to assess gene electrotransfer efficiency. The *pCIneo-DUX4* plasmid encoding the full length DUX4 protein is detailed in^110^. This plasmid contains the complete *DUX4 *ORF followed by the pLAM region isolated from the 4q35 fragment of a patient with FSHD. The *pCIneo* backbone vector from Promega (USA) was used as negative control. Expression plasmids used in this study are driven by cytomegalovirus (CMV) promoter. All DNA plasmids were produced by PlasmidFactory (Bielefeld, Germany) in a research grade quality for pre-clinical and veterinary studies.

### Intra-muscular injection and electroporation (hIMEP)

Adult female C57BL/6 mice were anesthetized by inhalation of 4% isoflurane in an induction chamber then maintained with 2% isoflurane using a mouse anesthesia mask. Hind limbs were shaved and 40 µg of hyaluronidase from bovine testes (Sigma-Aldrich, USA) diluted in 20 µl sterile saline buffer (0.9% NaCl, Physiodose, Gilbert Laboratories, France) were injected through the skin into the *tibialis anterior* (TA) muscle. Mice were then allowed to recover from anesthesia in their cages. After two hours, mice were re-anesthetized by an intra-peritoneal injection of Ketamine 100 mg/kg (Anestketin, Eurovet animal health) and Xylasine 10 mg/kg (Sigma-Aldrich, USA). Each TA was injected, with a Hamilton syringe, through the skin with one dose of naked pDNA (1, 5, 10, 20 or 40 µg) diluted in an equal volume of sterile saline buffer (50 µl). Flat parallel electrodes were placed on each side of the TA and good contact between electrodes and the overlying skin was ensured by use of a conductive gel (Rodisonic, Pannoc, Belgium). A train of eight square-wave pulses at a voltage of 95 V (200 V/cm; voltage to distance ratio calculated on the hind limb thickness mean) and a duration of 20 ms at 500 ms interval (2 Hz) was generated using an EMKA stimulator. After electroporation, animals were transferred back into their cages and were monitored until complete recovery from anesthesia. Mice were daily checked and then sacrificed by an intraperitoneal injection of Nembutal (120 mg/kg, CEVO) 1-, 3- or 7-days post electroporation. For vPMO preliminary test, both mouse TAs were electroporated with 20 µg of *pCIneo-DUX4* plasmid. Six hours after the procedure, mice received either an intra-peritoneal injection of 250 µg of vPMO pLAM3A − 12 + 13 (Gene tools, described in^54,72^) or no supplemental treatment. TA were harvested 7 days after hIMEP.

### Tissue preparation and histology

At the indicated euthanasia time points, right and left TAs were removed, embedded in OCT compound (VWR) and frozen in liquid nitrogen-cooled isopentane. Eight µm thick cryostat sections from proximal, medial and distal part of TA were cut using a Leica cryotome and serial sections were colored respectively with X-gal staining (β-gal staining kit, Invitrogen) to assess percentage and localization of muscle area expressing the electrotransfered genes, and with Hematoxylin–Eosin–Heidenhain blue (HEB) to evaluate the percentage of damaged muscle area. HEB coloration consists in a basic Hematoxylin–Eosin coloration followed by a 45-s incubation in Heidenhain’s Blue staining (mix of orange G and Aniline Blue, Sigma-Aldrich, USA), allowing an intense blue labeling of fibrotic fibresand collagenous tissues which improves contrast with healthy myofibres to facilitate muscle lesion quantifications. Slides were then scanned using the NanoZoomer-SQ Digital slide scanner (Hamamatsu Photonics). Images were processed by color thresholding using ImageJ 1.52a software, Rasband, W.S., ImageJ, U. S. National Institutes of Health, Bethesda, MA, USA, https://imagej.nih.gov/ij/, 1997–2018. The blue surface area was measured (threshold parameters: Hue = 35–255/50–255, saturation = 53–255/53–255 for X-gal/HEB staining respectively) and reported to the complete surface section. Measurements were performed on total section and on the injected area defined by X-gal staining (SI Fig. S3 online).

### Immunofluorescence

Tissue cryosections were fixed with 4% paraformaldehyde/PBS on ice for 20 min, permeabilized with 0.25% TritonX-100/PBS for 10 min, then incubated with blocking solution (5% normal goat serum (Dako), 2% BSA, 0.01% TritonX-100/PBS) for 30 min. Sections were then incubated with anti-DUX4 (rabbit monoclonal E5-5; 1:200, Abcam) and anti-laminin α (rat monoclonal; 1:100; Sigma-Aldrich) primary antibodies at 4 °C overnight. They were subsequently incubated with secondary antibodies Alexa 555 Goat anti-rabbit IgG (1:500, Biowest) and Alexa 488 Goat anti-rat Ig (1:500, Invitrogen) at room temperature for 1 h. Stained sections were mounted with EverBrite Mounting Medium with DAPI (Biotium) for nuclear staining. Pictures were taken with a Nikon Eclipse 80i microscope and merged using NIS-Elements software.

### RNA isolation, 3′RACE and real time quantitative PCR

#### RNA extraction

TA muscles were trimmed between proximal-medial and medial-distal parts using Leica cryotome (30 sections of 50 µm in both regions). These slices were ground into liquid nitrogen, homogenized in 1 ml of TRIzol reagent (Invitrogen) and RNA was isolated according to the manufacturer’s directions. Total RNA was then treated with DNAse I kit (amplification grade, ThermoFisher).

#### 3′RACE

cDNAs were synthetized using SuperScript III Reverse transcriptase kit (Invitrogen) and the 3′RACE adaptor of the RLM-RACE kit (5′-GCGAGCACAGAATTAATACGACTCACTATAGGTTTTTTTTTTTTN-3′, Ambion). The resulting cDNAs were amplified by nested PCR using PrimeSTAR Max DNA polymerase (Takara-bioJapan). The specific outer primer for *DUX4* cDNA amplification was: 5′-AGGCGCAACCTCTCCTAG AAAC-3′ and the inner primer was: 5′-TGGAAGCACCCCTCAGCGAGGAA-3′. Cycling conditions for outer PCR were as follows: initial denaturation step at 98 °C for 3 min, followed by amplification step with 25 cycles of 10 s at 98 °C, 10 s at 58 °C and 5 s at 72 °C, ended by 5 min at 72 °C. Same cycling conditions were used for inner PCR except the last step of the amplification which was 10 s at 72 °C instead of 5 s. The 3′RACE PCR products were analyzed by electrophoresis on 1%-agarose gel and staining with ethidium bromide (gel pictures provided Fig. 6 were made in compliance with the digital image and integrity policies of Scientific Reports). The products were then extracted from agarose band using PCR clean up gel extraction kit (Macherey–Nagel), cloned into pJET1.2/blunt (ThermoFisher), amplified in *E. coli* and sequenced (Genewiz Inc.) to confirm specific *DUX4* mRNA amplification and vPMO targeted sequence conservation.

#### RT-qPCR

cDNAs were synthetized using Maxima First Strand cDNA Synthesis kit (ThermoFisher). All qPCRs were performed in duplicates using SYBR Green FastStart Essential DNA Green Master (Roche) and following primers (10 µM): *Wfdc3* (5′-CTTCCATGTCAGGAGCTGTG-3′, 5′-ACCAGGATTCTGGGACATTG-3′) and *Zscan4c* (5′-GATTATTGGCCACAGGACAAG-3′, 5′-TCAGGGTGCTGTTCTTTCTG-3′) from^76^, *Myog* (5′-GAGACATCCCCCTATTTCTACCA-3′, 5′-GCTCAGTCCGCTCATAGCC-3′) and *Rplp0* (5′-TCATCCAGCAG GTGTTCG-3′, 5′-AGCAAGTGGGAAGGTGTAA-3′) custom designed (Eurogentec). Cycling conditions were as follows: initial denaturation step at 95 °C for 10 min, followed by 40 cycles of 15 s at 95 °C and 60 s at 60 °C. qPCR results were analysis with LightCycler 96 software (Roche).

### Statistical analyses

Statistical analyses were done using GraphPad Prism version 6.01 for Windows, GraphPad Prism software, La Jolla, CA, USA, www.graphpad.com. Data were tested for normality of distribution, using the Kolmogorov–Smirnov test, and all considered as non-parametric. Differences between experimental groups were statistically evaluated by a Mann Whitney rank sum test (hyaluronidase pre-injection, DUX4 expression and vPMO evaluation), one-way analysis of variance (ANOVA) on the ranks followed by a Dunn’s post hoc test for multiple comparison (dose–response, time–response and qPCR analysis), Friedman repeated ANOVA on the ranks (muscle distribution) or Wilcox on signed rank test (quantification on total vs injected area). Differences were considered statistically significant at a *p* value < 0.05. All data are represented as boxplot for non-parametric statistical tests (with minimum to maximum whiskers).

## Supplementary information

Supplementary file1 (PDF 1105 kb)
