# Supplementary material for: Induction of a local muscular dystrophy using electroporation in vivo: an easy tool for screening therapeutics
Source: Sci Rep. 2020 Jul 9;10:11301. doi: 10.1038/s41598-020-68135-7 (PMC7347864; doi:10.1038/s41598-020-68135-7)
Supplement: Supplementary file 1 — Supplementary file1 (PDF 1105 kb) [file 41598_2020_68135_MOESM1_ESM.pdf]

Supplementary information

Induction of a local muscular dystrophy using electroporation *in vivo*: an easy tool for screening therapeutics.

Aline Derenne[1,2*], Alexandra Tassin[2*], Thuy Hang Nguyen[2], Estelle De Roeck[2], Vincianne Jenart[2], Eugénie Ansseau[1], Alexandra Belayew[2], Frédérique Coppée[1], Anne-Emilie Declèves[1], and Alexandre Legrand[2£]

[1]Department of Metabolic and Molecular Biochemistry, Research Institute for Health Sciences and Technology, University of Mons, Mons, Belgium
[2]Department of Respiratory Physiology, Pathophysiology and Rehabilitation, Research Institute for Health Sciences and Technology, University of Mons, Mons, Belgium

* Both authors contributed equally to this study.
[£]Author to whom reprint requests should be addressed.

Email: alexandre.legrand@umons.ac.be

Supplementary Figure S1

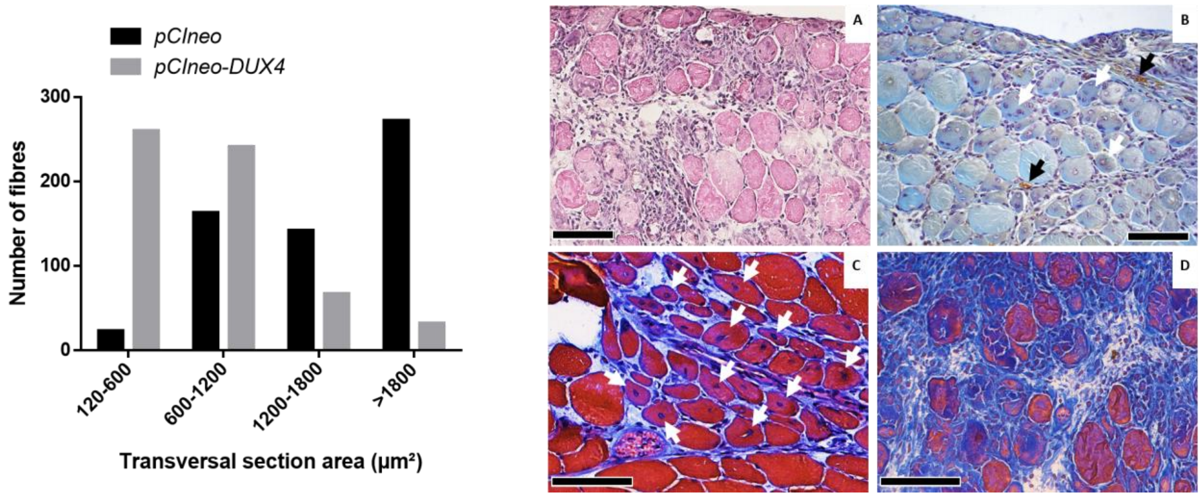

Figure S1. Characterization of DUX4-induced lesions in the hIMEP model. Left panel. *Morphometric analyses of TA myofibre transversal section area. Muscle sections of TA injected by hIMEP with 10µg pCMV-lacZ and 40µg of either pCIneo-DUX4 or pCIneo plasmid. Seven days post-injection, transversal cryosections were stained with HEB. The morphometric analysis of transversal section area of individual TA myofibres was performed using the image J software. A total of 600 myofibres selected in 3 randomly selected fields (per section) within the injected area were measured. Myofibres were then categorized in 4 size clusters. Fibre size distribution was represented as vertical bar chart histograms showing the number of fibres per size cluster (µm²). ****p<0,0001 pCIneo-DUX4 vs pCIneo, chi square.* ***Right panel.*** *Histological description of DUX4-induced lesions in the hIMEP model. Representative sections (medial region) of TA injected by hIMEP with 10 µg of pCMV-lacZ and 40 µg (A) or 1 µg (B, C, D) of pCIneo-DUX4. TA muscles were harvested one-week post injection.* ***(A)*** *Hematoxylin-Eosin staining highlights inflammatory infiltrates (nuclei stained in purple) and myofibres of varying size (in pink)* ***(B)*** *Immunohistochemistry using anti-F4/80 antibody (565409 BD Pharmingen; 1:50 dilution) shows the presence of macrophages colored in brown (black arrows). The Hemalun-Luxol Blue counter-coloration highlights delocalized nuclei (white arrows). Inflammatory infiltrates are also visible.* ***(C-D)*** *HEB staining reveals central nuclei (C, white arrows) and highlights conjunctive tissue accumulation (D, colored in blue). Scale = 100µm.*

Supplementary Figure S2

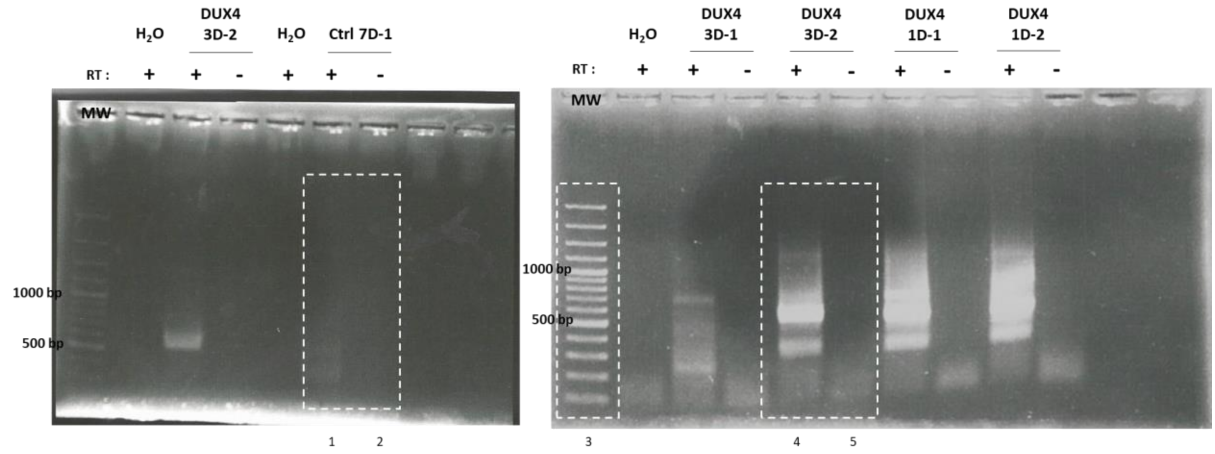

Figure S2. Confirmation of DUX4 mRNA expression by 3'RACE. *Nested PCR was used to amplify the 3' end of DUX4 transcripts from total RNA extracted from TA muscles electroporated with 1µg of pCIneo (Ctrl group) or pCIneo-DUX4 (DUX4 groups). 3'RACE was performed 1-, 3- or 7- days post hIMEP (1D, 3D and 7D, respectively). Wells (-) show negative controls without retro-transcription (RT). Boxes delineate representative cropped gel shown in the main text (figure 6).*

Supplementary Figure S3

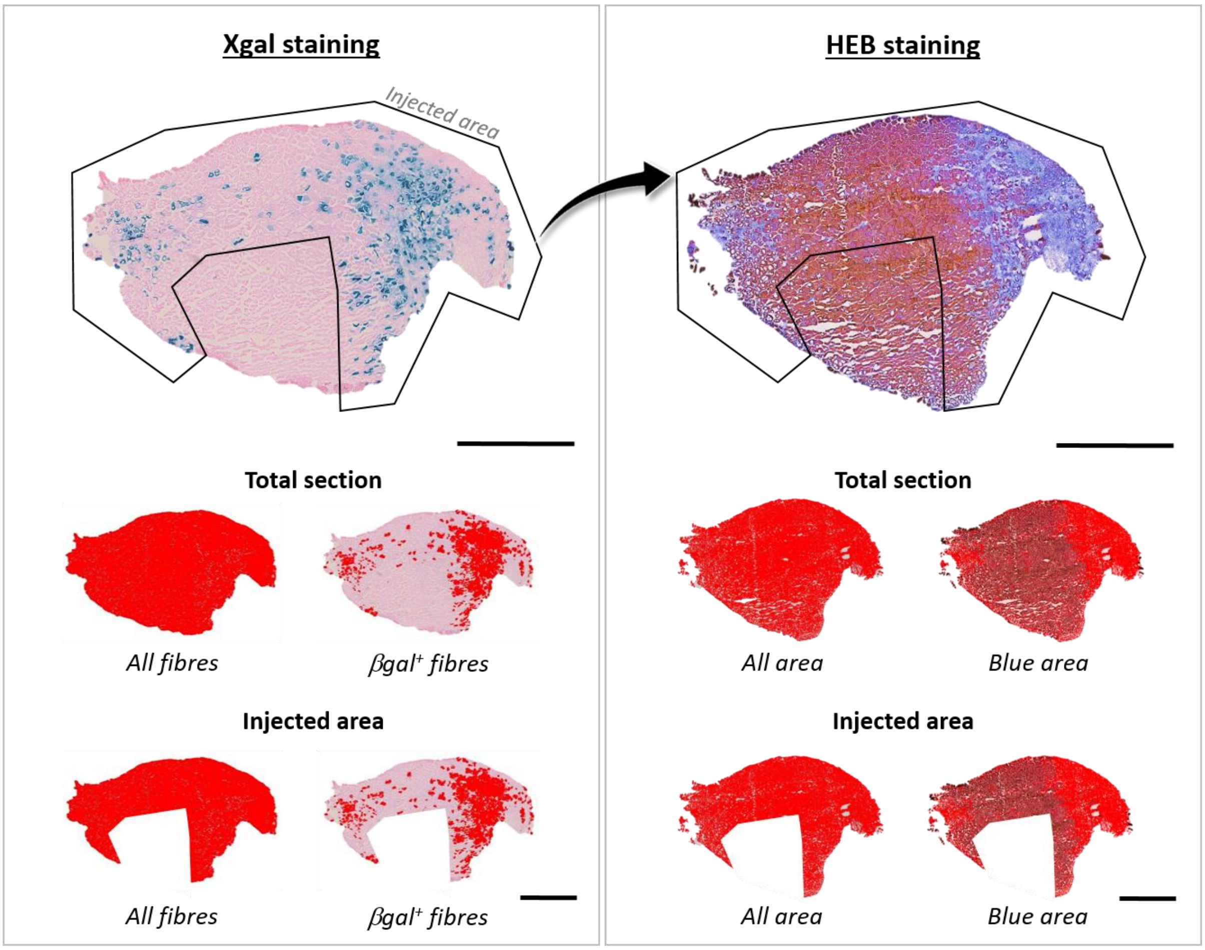

Figure S3. Surface quantification analyses : method. Left panel (Xgal staining). *Sections from proximal, medial and distal parts of TAs injected by hIMEP with 10 µg pCMV-lacZ were stained with X-gal to assess the percentage and the location of muscle area expressing β-galactosidase (in blue: β-gal^{+} fibres).* ***Right panel (HEB staining)****. Adjacent sections were stained with Hematoxylin-Eosin-Heidenhain blue (HEB) to evaluate the percentage of damaged muscle area (dark pink labeling of muscle fibres and blue labeling of fibrotic and collagenous tissues). Slides were scanned using the NanoZoomer-SQ Digital slide scanner (Hamamatsu Photonics) and images were processed by color thresholding using ImageJ 1.52a software. The blue surface area was measured (threshold parameters: Hue=35-255/50-255, saturation=53-255/53-255 for X-gal/HEB staining, respectively) and reported to the section surface. Measurements were performed on total section and on the injected area defined on the basis of the X-gal staining. Measured surface after thresholding is shown in red on the small pictures. Scale = 1mm.*
